# Utility of Multiparametric Prostate MRI to Predict Regional or Distant Metastatic Disease Against Conventional Staging Using CT and Bone Scintigraphy or 68Ga-PSMA PET in Intermediate-to-High-Risk Prostate Cancer

**DOI:** 10.7759/cureus.73607

**Published:** 2024-11-13

**Authors:** Minhtuan Nguyenhuy, Xiang Qian Chan, David Homewood, Cindy Ogluszko, Philip Dundee, Niall Corcoran

**Affiliations:** 1 Department of Radiology, Austin Health, Melbourne, AUS; 2 Department of Urology, Western Health, Melbourne, AUS; 3 Department of Urology, Royal Melbourne Hospital, Melbourne, AUS

**Keywords:** diagnostic accuracy, ga68 psma pet/ct, prostate cancer, prostate mri, tumour staging

## Abstract

Background: Multiparametric magnetic resonance imaging (mpMRI) is now the standard of care to guide prostate biopsies during workups and assessment of men with suspected prostate cancer (PCa). In addition to intraprostatic lesion detection, MRI usually covers the bony pelvis and pelvic lymph nodes, two of the commonest sites for metastatic disease. Subsequent staging has traditionally been based on further scanning using a combination of computed tomography (CT) and bone scintigraphy (BS), and more recently, positron emission tomography (PET) scanning with prostate-specific membrane antigen (PSMA) ligand. However, the value of additional staging investigations for men who are negative for metastatic disease on pelvic MRI is unclear. This study aims to evaluate the concordance of MRI findings with other imaging performed during staging.

Methods: Patients with a Gleason score (GS) of ≥ 7 who had received both a pre-biopsy mpMRI and subsequent staging investigations from a single institution between 2019 to 2022 were identified. Imaging reports for PET, CT, and BS were used as the reference standard to evaluate MRI accuracy. PSMA-PET was considered the definitive outcome if multiple scans were performed. MRI findings were then classified as positive, negative, or equivocal. The accuracy was calculated using interpretations where equivocal cases were considered positive or negative for spread, representing a ‘pessimistic’ or ‘optimistic’ reading, respectively. A subgroup assessment of results considering only the use of CT + BS and PET was also done.

Results: This study identified 214 patients for inclusion. The median age was 70 (IQR: 65-75) years, prostate-specific antigen (PSA) was 9.65 (IQR: 6.9-14.3) (ng/ml), and PSA density was 0.26 (ng/ml/cc) (IQR: 0.15-0.46). Complete conventional staging was performed for 130 patients, and PSMA-PET was performed for 102 patients. The results for the optimistic against pessimistic interpretations were the following: overall accuracy (90% vs 89%), sensitivity (0.48 vs 0.52), specificity (0.97 vs 0.95), negative predictive value (NPV) (0.84 vs 0.93), and positive predictive value (PPV) (0.71 vs 0.63). When comparing subgroup results considering only conventional imaging against only PSMA-PET, there were markedly more discordant findings in the PET group.

Conclusion: The impression of nodal and metastatic status through mpMRI poorly correlates with results from conventional staging and PSMA-PET. PSMA-PET more often produces discordant results to mpMRI, signifying an additive diagnostic value. MRI should not be used alone in the workup of prostate cancer in patients with a GS ≥ 7, where metastasis is a concern.

## Introduction

Prostate cancer (PCa) is the second-leading cause of cancer-related mortality in the United States among males and is the most frequently diagnosed tumour in this population [1]. In those with organ-confined disease, definitive treatment with radical prostatectomy or radiotherapy can improve patient survival. Historically, PCa has been diagnosed via an ultrasound-guided prostate biopsy in those with an elevated prostate-specific antigen (PSA) level or an abnormal digital rectal examination, with subsequent staging performed using a combination of CT and MRI to assess any locoregional or visceral disease and addition of bone scintigraphy (BS) to assess for any osseous metastasis. A prostate-specific membrane antigen (PSMA) positron emission tomography (PET) scan is an alternative method of staging for select high-risk patients or those with equivocal findings.

Recent studies have suggested that performing an MRI prior to a biopsy increases diagnostic targeting and histopathological assessment resulting in reduced overdiagnosis and improved management in those with Pca [2-4]. Consequently, the routine use of mpMRI prior to initial prostate biopsies has been endorsed by several urological societies reflecting a shift in the standard management of PCa [5]. Importantly, research has shown that PCa metastatic spread follows a predictable pattern with the initial involvement of pelvic structures within the mpMRI field of view. Pelvic lymph node metastases almost always precede distal nodal involvement, and osseous metastases have been shown to initially involve the pelvis and lumbar vertebrae [6,7]. This characteristic pattern of spread suggests that a negative mpMRI may be sufficient for a locoregional assessment, warranting a re-evaluation of the necessity and utility any additional staging scans may provide.

Prior analysis has suggested non-inferior performance of MRI in comparison with conventional CT and BS in evaluating nodal and bone involvement [8,9]. Additional advancements and standardisation of imaging protocols advised in recent editions of the Prostate Imaging Reporting and Data System (PI-RADS) scoring system (American College of Radiology (ACR), Reston, Virginia, United States; European Society of Uroradiology (ESUR), Copenhagen, Denmark; and AdMetech Foundation, Boston, Massachusetts, United States), such as recommendations for acquisition using 3T over 1.5T MRI, are also avenues for improved diagnostic accuracy [10-12]. While more recent advances in medical imaging have indicated that the use of PSMA-PET CT demonstrates improved diagnostic accuracy over conventional staging, accessibility and resource availability are constraining factors in widespread adoption at present [13]. The use of either imaging modality for staging is considered acceptable within oncological tumour boards to guide the management of PCa.

Oncological outcomes in PCa are widely understood to significantly differ based on tumour biology. The Gleason score (GS) pattern is one such grading system used for PCa. Low-risk Gleason 6 PCas, while harbouring cytological hallmarks of cancer, have a clinical course with negligible metastatic potential. Comparatively, GSs of 7 and 8-10 indicate intermediate and high-risk PCas, respectively, reflecting a significant metastatic potential [14,15]. Prior evaluations assessing the accuracy of prostate mpMRI have often included patients with GS 6 disease, which is a source of potential bias, due to a higher proportion of true negative results [16].

This study aims to evaluate the diagnostic accuracy of prostate mpMRI against conventional staging with CT and bone scans or PSMA-PET CT to detect nodal and bony metastasis in patients with histopathologically confirmed GS ≥ 7 PCa to assess the added benefit of additional imaging.

## Materials and methods

Patients with histopathologically confirmed GS ≥ 7 PCa who underwent both mpMRI and conventional staging with CT and BS with or without PSMA-PET scan were retrospectively identified from databases affiliated with a single tertiary institution (Western Health, Melbourne, Australia). Patient inclusion was constrained to only those who had undergone MRI after 2019 to reflect the standardisation of MRI acquisition protocols introduced in the PI-RADS version 2.1 (v2.1) system. Patients were enrolled from January 2019 until December 2022. Exclusion criteria were the following: (1) synchronous cancer, (2) elapsed date ≥ six months between diagnostic imaging, (3) scans performed to assess treatment response, and (4) non-diagnostic imaging. Baseline characteristics were recorded including the age at the time of the mpMRI staging, including PSA, prostate volume, PSA density, PI-RADS score, and GS, which was then converted into the International Society of Urological Pathology (ISUP) grade for PCa.

Representative imaging and protocol details included the following: (1) 3.0T MRI, Axial T1 whole pelvis, and high-resolution sagittal, axial, and coronal T2 images, diffusion-weighted imaging (DWI), apparent diffusion coefficient (ADC) maps (gradient B values: 50, 400, 800, and 1500 second/mm^2^), IV gadolinium contrast, and hyoscine butylbromide administration; (2) BS with ligand technetium-99 metastable (99mTc) hydroxymethylene diphosphonate (HDP), single-photon emission CT (SPECT)-CT with a field of view (FOV) covering the chest to the pelvis; (3) PSMA-PET: Siemens 64-slice time of flight (TOF) PET/CT (Siemens Healthineers, Erlangen, Germany) with ligand 18F-PSMA with contrast enhancement; (4) Staging CT: Pre- and post-contrast-enhanced CT with a FOV covering the chest, abdomen, and pelvis.

This study was granted ethics approval from the Western Health Low-Risk Human Research Ethics Panel (approval number: HREC/23/WH/95356).

Overview of imaging, histopathology, and treatment protocol

All MRI scans performed were conducted in accordance with PI-RADS v2.1 recommendations. Conventional staging was the default assessment for patients until July 2022, when governmental reimbursement was approved nationally in Australia for the performance of PSMA-PET during the initial assessment of patients with GS ≥ 7. Prior to this, PSMA-PET was reserved for those considered high-risk for extracapsular extension and metastatic dissemination or for those with equivocal findings on conventional staging. All medical imaging was interpreted by board-qualified radiologists to identify the presence of nodal and osseous metastasis. Lymph nodes were considered positive in MRI and CT based on the radiologist's impression describing the following: (1) any suspicious node for metastases, (2) abnormal morphology or irregular margins, or (3) > 8 mm in short axis. Equivocal findings on MRI were considered if there was any description of an indeterminate or equivocal appearance. Positive cases on PET scans were determined by any description of significant PSMA avidity consistent with metastasis. Bone metastasis on all other modalities was considered positive if there were any changes considered attributable to metastasis that was not benign.

Results were trichotomized into normal, metastatic, and equivocal for MRI findings, while conventional staging and PSMA-PET were considered the gold standard and dichotomised into normal and metastatic findings. In cases of discordance between conventional staging and PET, the latter result was used as the definitive outcome.

All prostate biopsies were attained using guidance facilitated by pre-procedural prostate mpMRI. Sampling was performed with either trans-perineal systemic sampling or trans-perineal PrecisionPoint biopsy (Perineologic, Cumberland, Maryland, United States) with MRI-directed cognitive or electronic image fusion. Histopathological specimens were reported using the GS system and tiered ISUP grade groups according to the latest guidelines by board-qualified pathologists.

Statistics

Statistical analyses were performed using Microsoft Excel version 2306 (Microsoft Corporation, Washington, United States). Continuous variables were evaluated as median and IQR. Binary variables are presented as incidence as a percentage. Outcomes relating to metastatic findings were evaluated using 2 x 2 contingency tables to determine diagnostic accuracy relating to sensitivity, specificity, positive predictive value (PPV) and negative predictive value (NPV). PSMA-PET was used as the definitive outcome, followed by CT + BS results. Analysis was performed twice with optimistic and pessimistic interpretations for equivocal outcomes on MRI. In the optimistic scenario, any equivocal findings were relabelled as normal, and in the pessimistic scenario, equivocal findings were labelled as metastatic.

A subgroup evaluation of diagnostic accuracy was performed for cohorts in which only conventional staging or PSMA-PET was completed. In the conventional staging subgroup, any results from PSMA-PET were blinded.

## Results

Overall, there were 214 patients identified with newly diagnosed PCa who underwent pre-biopsy mpMRI with subsequent complete staging (Table 1). In total, there were 130 patients who received complete conventional staging, 102 who received PSMA-PET, and 18 patients who received all imaging. The median age was 70 (IQR: 65-75) years, PSA was 9.65 ng/ml (IQR: 6.9-14.3), and PSA density was 0.26 ng/ml/cc (IQR: 0.15-0.46). The distribution of ISUP grade groups and PIRADS lesions is presented in Table 1. The most common PI-RADS score was 5 (59%), and the most common ISUP grade was 3 (29%).

**Table 1:** Patient demographics IQR: interquartile range; PI-RADS: Prostate Imaging Reporting and Data Systems; ISUP: International Society of Urological Pathology; PSA: prostate-specific antigen; CT + BS: computed tomography (CT) and bone scintigraphy; PSMA-PET: prostate-specific membrane antigen-positron emission tomography

Parameter	Patients
Total patients	214
Age (year), median (IQR)	70 (65-75)
PSA (ng/ml), median (IQR)	9.7 (6.9-14.3)
Prostate volume (cc), median (IQR)	39 (27-51.5)
PSA density (ng/ml/cc), median (IQR)	0.26 (0.15-0.46)
PI-RADS V2 grade	
PI-RADS 5	126 (59%)
PI-RADS 4	57 (27%)
PI-RADS 3	18 (8%)
PI-RADS 2	12 (6%)
PI-RADS 1	0 (0%)
ISUP grade group	
ISUP 5	52 (24%)
ISUP 4	28 (13%)
ISUP 3	62 (29%)
ISUP 2	71 (33%)
MRI performed	214 (100%)
CT + BS performed	130 (61%)
PSMA-PET performed	102 (47%)
Equivocal readings	9 (4%)

In total, there were 40 (19%) patients identified with lymph node involvement and 18 (8%) with osseous lesions consistent with metastases. There were only 9 (4%) equivocal readings overall. Consequently, regardless of a pessimistic or optimistic interpretation, overall accuracy metrics were similar. PSMA-PET demonstrated higher detection rates for nodal (25% vs 15%) and osseous (10% vs 8%) metastases compared to conventional staging.

There was a high concordance between MRI and other forms of staging with a 90% agreement rate. For any finding, the specificity (0.95-0.97) and NPV (0.84-0.93) were high, contrasting with lower sensitivity (0.48-0.52) and PPV (0.63-0.71) (Table 2).

**Table 2:** Diagnostic accuracy of MRI MRI: magnetic resonance imaging; CT: computed tomography; BS: bone scintigraphy; NPV: negative predictive value; PPV: positive predictive value

Diagnostic accuracy						
Optimistic interpretation	Cases	Accuracy	Sensitivity	Specificity	NPV	PPV
MRI vs Any staging: Any finding	428	387 (90%)	0.48	0.97	0.84	0.71
MRI vs Any staging: Nodal	214	187 (87%)	0.48	0.97	0.89	0.62
MRI vs Any staging: Osseous	214	200 (93%)	0.5	0.97	0.96	0.64
MRI vs CT / BS: Nodal	130	119 (92%)	0.68	0.95	0.95	0.72
MRI vs CT / BS: Osseous	130	124 (95%)	0.7	0.98	0.98	0.7
MRI vs PET: Nodal	102	83 (81%)	0.36	0.96	0.82	0.75
MRI vs PET: Osseous	102	91 (89%)	0.2	0.97	0.92	0.4
Pessimistic interpretation	Cases	Accuracy	Sensitivity	Specificity	NPV	PPV
MRI vs Any staging: Any finding	428	382 (89%)	0.52	0.95	0.93	0.63
MRI vs Any staging: Nodal	214	182 (85%)	0.53	0.93	0.89	0.62
MRI vs Any staging: Osseous	214	200 (93%)	0.5	0.97	0.96	0.64
MRI vs CT / BS: Nodal	130	115 (88%)	0.68	0.92	0.94	0.59
MRI vs CT / BS: Osseous	130	124 (95%)	0.7	0.98	0.98	0.7
MRI vs PET: Nodal	102	81 (79%)	0.44	0.91	0.83	0.61
MRI vs PET: Osseous	102	91 (89%)	0.2	0.97	0.92	0.4

In analysing the low sensitivity values, subgroup assessment of nodal accuracy showed an increased sensitivity when only using conventional staging as a comparator, and conversely, a decreased sensitivity when PET was the only comparator (any staging: 0.48-0.53, CT: 0.68, PET: 0.36-0.44). Osseous involvement demonstrated a similar trend, with worse accuracy metrics when compared with PET only (any staging: 0.5, CT + BS: 0.7, PET: 0.2). 

The accuracy of mpMRI was overall similar regardless of whether bone or nodal involvement was considered, although mpMRI to evaluate bone metastasis appears particularly deficient when compared with PET.

There were only 18 patients who received conventional staging and PET, which limited the ability to directly compare the performance between these two modalities.

## Discussion

Accurate staging is fundamental in directing the treatment of PCa patients; however, this must be balanced against limited healthcare resources. Recent changes in PCa management with the routine use of mpMRI during initial pre-biopsy workup therefore require a re-evaluation of the benefit provided by any additional imaging. In this cohort of patients with intermediate to high-risk PCa, we found that for the assessment of nodal and osseous metastasis, while the overall accuracy, specificity, and NPV of MRI were high, there was a comparatively lower sensitivity and PPV. Furthermore, subgroup assessment of MRI results when considering only CT + BS for adjunctive staging showed a sensitivity of 0.68-0.70, which decreased to 0.2-0.44 when PSMA-PET data were considered. This reflects a higher congruence of MRI with CT + BS over PSMA. As such, these findings suggest that mpMRI alone to detect metastasis for locoregional staging is not advisable, with further implications that even the use of CT + BS, which remains accepted in many clinical pathways, may lack the ability to detect subtle or occult metastasis when compared with PSMA-PET.

It is important to note that these findings compare the accuracy of mpMRI against other imaging modalities rather than histological assessment with pelvic-lymph-node dissection (PLND). While less accurate, this is important as it reflects the trend towards modern evaluation of local staging. Multiple studies have shown no improvement in patient biochemical-free or overall survival in those undergoing PLND at the time of radical prostatectomy (RP), with a decline in nodal dissections performed over the past decade [17-20]. Although histological confirmation through PLND has traditionally been considered the gold standard, its clinical utility has been increasingly questioned. Accurate staging remains crucial for guiding treatment intensification with hormonal therapy, chemotherapy, and radiotherapy where necessary. Despite using imaging as the gold standard which would over-estimate the true accuracy of mpMRI, this approach provides an important measure against current PCa management.

There are limited modern assessments comparing mpMRI against other diagnostic imaging modalities in the literature. Previously, Woo et al. reported a substantially higher sensitivity of 0.95 for mpMRI in detecting bone metastasis on a per-patient level [21]. However, their study preceded the use of PSMA-PET and did not specify imaging for comparison. Further, it was unclear if the determination of MRI findings was interpreted retrospectively or prospectively. Similarly, Meissner et al., directly compared the performance of 3T MRI against PSMA-PET as the reference standard, suggesting an MRI sensitivity and PPV of 0.82 and 0.74, respectively; however, limited nodes to below the aortic bifurcation and evaluated performance through retrospective double-reporter reading [22]. In another meta-analysis, while CT and MRI were suggested to have comparable ability to detect positive nodes, many of the articles comprising this assessment were performed over two decades ago, with limited relevance to modern diagnostic imaging [9]. A unique aspect of our study is that these results in the present study have been determined from patient records guiding treatment, rather than re-evaluations of medical imaging, which reduces observer bias inherent to retrospective analyses. These results may more accurately reflect accuracy in the clinical setting.

A key limitation of CT and MRI in identifying pathological nodes is that the assessment is often determined by the following morphological criteria: size (short-axis diameter > 8 mm), the presence of irregular margins, or hypodensities representing necrosis, but it is not uncommon for morphologically normal nodes to harbour metastatic disease. MRI has been suggested to have some advantages over CT in this aspect, as nodal involvement has been characterised by diffusion restriction, resulting in a high DWI and low ADC signal, owing to increased cellularity restricting the anisotropic movement of water [23]. Despite these advantages, overlaps in ADC and DWI values between benign and malignant nodes limit the consistent reliability. As an additive tool, Hotker et al. have suggested that the prediction of lymph node invasion (LNI) be improved when combining ADC values to the Briganti nomogram for positive nodes [24].

While it is possible that the reduced sensitivity of MRI compared with CT may be attributable to improved lymph node evaluation, this is unlikely as this should lead to an increased sensitivity, whereas a decrease in sensitivity was actually observed. The major challenge in interpreting bone lesions detected on MRI is that the pelvic bones are usually only seen on large FOV T1-weighted images, and as such, are incompletely assessed without additional MRI sequences such as those with fat-suppression techniques [25]. This poses a significant challenge in differentiating between benign and malignant bone lesions. Radiologists may be less inclined to make definitive statements about bone lesions at the initial imaging, knowing that patients with positive prostate biopsies will undergo further dedicated imaging.

Limitations

This single-institution study has inherent limitations in generalizability, particularly given the institutional approach to PCa management. The patient workup followed a structured pathway determined by the national funding criteria (Australian Medical Benefits Schedule). Initially, the patients qualified for mpMRI to guide transperineal prostate biopsy, consistent with the Prostate MRI Study (PROMIS) trial recommendations [26]. Subsequently, those with clinically significant prostate cancer (GS ≥ 7) would undergo further workup with conventional staging with CT and bone scintigraphy. PSMA-PET funding became available during the latter half of the study period, introducing an additional staging option. This may have influenced radiology reporting patterns, as the primary focus was on the accurate assessment of prostatic lesions amenable to biopsy on MRI and to evaluate features not assessable by other cross-sectional imaging findings such as PI-RADS grade, extra-prostatic extension, and perineural invasion, as those with confirmed cancer would nonetheless undergo further dedicated imaging. The awareness of subsequent imaging to further characterise nodal and bone involvement, and underlying expectations regarding the accuracy of MRI for these findings may have influenced the approach to interpreting studies.

There are several important limitations to our study. Primarily, this study retrospectively collected data from radiology impressions in the prospective clinical setting rather than quantify the accuracy of MRI using a multi-reader diagnostic accuracy study design. While this may limit the theoretical accuracy of MRI, it also is a strength in that it reflects real-world clinical accuracy if referring clinicians are to solely rely on an initial pre-biopsy MRI to guide local staging. Another limitation is that the data were not labelled to indicate the specific location of any findings, so it is unclear if these results would have been within the MRI FOV, and consequently, the accuracy on a per-lesion basis remains unclear. This study was conducted within a single institution, and any subsequent local staging with CT + BS would have benefitted from correlation with pre-existing MRI results to inform subsequent radiological interpretation.

## Conclusions

In the clinical setting, MRI performed to guide prostate biopsy poorly correlates with results from conventional staging with CT and bone scans, and neither with PSMA-PET. PSMA-PET is more often discordant with MRI when compared with conventional staging, signifying an additive diagnostic value. If resource availability or diagnostic uncertainty exists, a PET scan should be favoured during staging. Localised mpMRI alone should not be used by itself in the workup of patients with PCa with GS ≥ 7, where there is a clinically significant suspicion of metastatic disease. 
